# Displacement of mining vibrating screen obtained from acceleration based on improved S–G filter

**DOI:** 10.1038/s41598-024-53823-5

**Published:** 2024-02-07

**Authors:** Linjing Xiao, Hao Lu, Fangping Yan

**Affiliations:** 1 Department of Electrical and Information, Shandong University of Science and Technology, Jinan, 250031 Shandong China; 2 College of Mechanical and Electronic Engineering, Shandong University of Science and Technology, Qingdao, 266590 China

**Keywords:** Vibration displacement, Acceleration signals, Improved Savitzky–Golay filter, Particle Swarm Optimization algorithm, Frequency-domain integration, Mechanical engineering, Information technology

## Abstract

Vibration displacement is one of the key parameters in fault diagnosis of vibrating screens. Monitoring of acceleration signals of vibrating screens can be disturbed due to various factors such as on-site working conditions and equipment. In order to obtain accurate displacement signals of vibrating screen, the method for converting vibration acceleration to displacement based on improved Savitzky–Golay (S–G) filter is proposed. The Particle Swarm Optimization (PSO) algorithm is used to optimize the window length of the S–G filter with the fixed polynomial. The filters are cascaded to denoise the signals multiple times. The reasonable regularization parameter of the Smoothed Prior Approach (SPA) is calculated to remove the trend item from the acceleration signals. The vibration displacement is obtained by integrating the preprocessed acceleration data in the frequency domain. The results demonstrate that the objectivity of parameter selection of filter is improved, and the denoising effect is significant. The filtering effect of the filter is further improved after cascading. It becomes better as the number of stages of cascade increases. The vibration displacement can be obtained accurately by the proposed method. The vibration test platform is built to verify the correctness of the method.

## Introduction

Vibration displacement is an important basis to analyze the working condition and fault diagnosis of vibrating screen. The acceleration signals contain a large amount of interference due to the complex working conditions. Significant errors can be caused if the acceleration signals are directly converted into displacement signals by the integral method^1^. Thus, the acceleration signals should be preprocessed to obtain the displacement of the vibrating screen.

Filtering is one of the effective methods for removing noise. Lotfi et al. used smoothing based on the diffusion equation for denoising to reduce the integral drift. However, the method relied on the local characteristics of the signals, such as gradient and curvature, and could not adaptively adjust the smoothing parameters according to the local characteristics of the signals^2^. Guo et al. denoised acceleration signals of ship equipment using median filtering. But the median filtering caused information loss and drift of acceleration integration^3^. Natalia et al. proposed a novel procedure for technical condition monitoring of a vibrating screen in the presence of impulsive noise. Wavelet filtering was used for noise reduction. However, there were different effects with the different choices of parameters, such as wavelet basis function and decomposition level. The wavelet filtering exhibited a certain degree of subjectivity in denoising^4^. Dombi et al. proposed an adaptive design method for signal smoothing based on the Savitzky–Golay (S–G) filter to solve the problem of poor performance of S–G filter in processing signals with a high rate of change. The denoising effect of the method was significant, and the applicability was verified by simulation^5^. Tanu et al. proposed an S–G filter cascade method in order to improve the denoising effect of the S–G filter. The appropriate parameters of the filter were determined based on the correlation coefficient and signal-to-noise ratio (SNR) to design filters with different cascading methods. The filtering effect of the S–G filter cascade was more significant than that of a single stage, but the selection of its parameters was highly subjective^6^. Liu et al. used the cuckoo algorithm to optimize the parameters of the S–G filter, which took the maximization of the gray correlation between the original signals and the denoised signals as the optimization objective. The effect of the S–G filter was improved, however, the search vitality of the cuckoo algorithm was insufficient, resulting in a slow filtering speed^7^.

The trend item will affect the accuracy of the collected acceleration signals^8^. Gao et al. analyzed the effect of the least squares method for removing the trend item from signals. This method made it easy to eliminate a large amount of helpful information for both random and periodic signals under short sample length, which caused the distortion in the displacement curve obtained by integration^9^. Zhang et al. used Empirical Mode Decomposition (EMD) to separate the trend item from the acceleration signals. However, the denoising performance of the EMD was seen as less satisfactory for linear and stationary interference. Furthermore, it was essential to depend on experience when selecting appropriate termination conditions and determining the degree of decomposition^10^. Miao et al. used a digital filter to eliminate the trend item in the acceleration signals to solve the problem of baseline drift in acceleration integration. The proposed method was verified compared with the low-frequency cutoff and low-frequency attenuation methods, but it caused the phase delay phenomenon^11^. Wu et al. proposed a segmented integration method to remove the trend item from long-term vibration signals, which was difficult to remove. However, it was essential to depend on experience when determining the number of segments. The more segments the signal was divided into, the more detailed the trend changes were captured, which also led to an increase in computational complexity^12^. Yu et al. proposed a method for removing the trend item based on the Smoothed Prior Approach (SPA) for the pre-assumption of the type of the trend item and computational complexity in the existing methods. The cutoff frequencies of SPA under different regularization parameters were analyzed, and the regularization parameters were obtained based on the frequency range of the trend item in the target acoustic signals to remove the trend item in the signals effectively^13^. Su et al. proposed a smoothing prior method based on regularized least squares to remove the trend item of pulse waves. Compared with Wavelet Transform and EMD, this method had a better effect on removing the trend item and calculation speed^14^.

Liu et al. have developed a program using LabVIEW to study the problem of the vibrating amplitude of each spring seat of the mining vibrating screen being different. The displacement signals are calculated from acceleration signals of the mining vibrating screen after high-pass filtering, wavelet-analysis detrend and trapezoidal integral^15^. Li changed the vibration displacement of the mining vibration-dewatering screen by adjusting the angle between the two eccentric blocks inside the vibration motor, drew the motion track of the mining vibration-dewatering screen through cardboard and pencil, and measured vibration displacement using a vernier caliper^16^. Fang et al. obtained the displacement signals from the collected acceleration signals of the mining vibrating screen through frequency domain integration based on FFT filtering (FFT-FDI)^17^. Linhares et al. wrote the program in Catman Esay AP software to obtain the displacement data. In this program, the acceleration signals were double integrated with the assistance of a Bessel frequency filter to obtain the displacement signals^18^.

To sum up, the problems of insufficient interference removal and strong subjectivity exist in the current methods used in processing acceleration signals, which affect the accuracy of the obtained displacement signals. In this paper, the method for converting vibration acceleration to displacement based on an improved S–G filter was proposed. The particle swarm algorithm was used to optimize the window length of the S–G filter to reduce human intervention. The cascaded optimized filter and SPA were combined to remove the interference more significant. Finally, the accurate displacement was obtained through frequency-domain integration.

## Acceleration signals preprocessing

### Signals composition

The actual acceleration signals of the mining vibrating screen inevitably have interference terms, including random interference and trend item, and it can be expressed as^19^:

1 $$ {\text{a}}_{{m}} {(}t{)} = a_{{r}} {(}t{)} + {\text{a}}_{{n}} {(}t{)} + {\text{a}}_{t} {(}t{),} $$

where *t* is time, ${\text{a}}_{r} {(}{\text{t}} {)}$ is ideal acceleration signal, ${\text{a}}_{{n}} {(}{\text{t}} {)}$ is random interference, ${\text{a}}_{t} {(}t{)}$ is trend item.

The displacement obtained by double integrating the acceleration in the time domain can be expressed as:

2 $$ {\text{x}} {(}t{)} = \iint {a_{{r}} }{(}t{)}dtdt + \iint {a_{{n}} }{(}t{)}dtdt + \iint {a_{t} }{(}t{)}dtdt + \varsigma t + v_{{0}} t + \tau + x_{{0}} , $$

where $\tau$ and $\varsigma$ are constants, ${\text{x}}_{0}$ and ${\text{v}}_{0}$ are actual initial displacement and initial velocity, respectively.

The displacement can be divided into three parts, which are ideal displacement signal, interference caused by ambient noise, and trend item as follows:

3 $$ {\text{x}}_{{1}} {(}t{)} = \iint {a_{{r}} {(}t{)}dtdt}, $$

4 $$ {\text{x}}_{{2}} {(}t{)} = \iint {a_{{n}} {(}t{)}dtdt}, $$

5 $$ {\text{x}}_{{3}} {(}t{)} = \iint {a_{t} }{(}t{)}dtdt + \varsigma t + v_{{0}} t + \tau + x_{{0}} . $$

### Noise removal

#### S–G filter

S–G filter is widely used for smoothing data streams. The local smoothing signals are obtained by fitting the specified polynomial to the sample of the symmetric window using the least squares method^20^. The shape and width of the original signals are maintained while the noise is effectively removed^21^.

It is assumed that the window length of the S–G filter is 2*n* + 1, and the data to be fitted is ${\text{a}} = \left\{ { - n{,} - n + {1,}L{,0,1,}L{,}n - {1,}n} \right\}$. The data within the window is fitted by an *n*th-order polynomial, which is expressed as^22^:

6 $$ {\text{f}} {(}a{)} = b_{{0}} + b_{{1}} a + b_{{2}} a^{{2}} + \cdots + b_{{k - {1}}} a^{{k - {1}}} , $$

where *a* is the data to be fitted, ${\text{b}}_{i} {(}{\text{i}} \in {[}0{,}{\text{k}} - 1{])}$ is coefficient.

The fitting equation can be expressed in matrix form as follows:

7 $$ {\text{Y}}_{{{(2}{\varvec{n}} + {1)} \times {1}}} = A_{{{(2}{\varvec{n}} + {1)} \times {\varvec{k}}}} \times B_{{{\varvec{k}} \times {1}}} + E_{{{(2}{\varvec{n}} + {1)} \times {1}}} , $$

where $A$ is data value matrix, $B$ coefficient vector and $E$ is residual vector.

The filtered signals are expressed as:

8 $$ \mathop {\text{Y}} \limits^{ \wedge } = {\text{A}} \cdot {\text{B}} . $$

**Figure 1:**
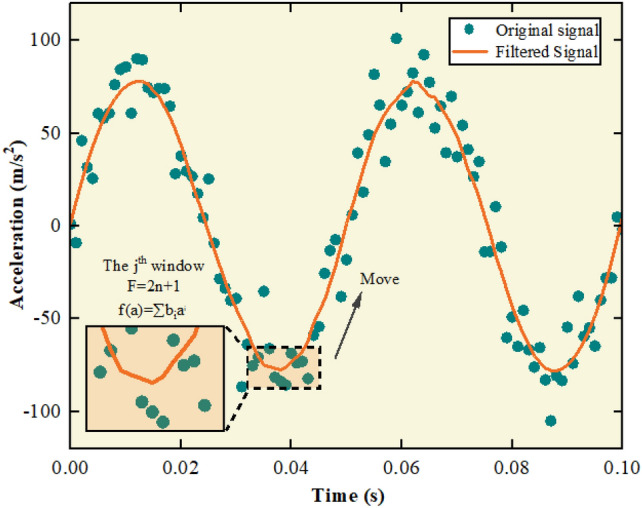
Schematic diagram of S–G filtering.

The schematic diagram of the S–G filter is shown in Fig. 1.

Appropriate window length and polynomial order can not only improve denoising effect and reduce signal distortion, but also improve filtering efficiency. It is necessary to optimize the S–G filter to improve the filtering effect. There are two optimization strategies: one is to fix the window length and optimize the polynomial order, and the other is to fix the polynomial order and optimize the window length. Using the longer window length effectively reduces noise but may lose signal details, while the shorter window length preserves details but reduces less noise. The accuracy of the processed signals can hardly be ensured when the window length is fixed. In addition, the calculation time is determined by the order of polynomials. The computational efficiency decreases as the order increases. The accuracy is more critical than the computational efficiency. Therefore, the second optimization strategy is adopted in this paper.

#### S–G filter cascade based on Particle Swarm Optimization algorithm (PSO-CSG)

Particle Swarm Optimization (PSO) is a swarm intelligence parallel optimization search method inspired by the foraging behavior of bird flocks^23^. It has a fast convergence speed and few parameters.

It is assumed that a swarm is composed of *M* particles in the *D*-dimensional target search space. The position and velocity of the *i*th particle are expressed as:

9 $$ {\varvec{X}}_{id} = \left[ {{\text{x}}_{{{\text{i}} 1}} {,}{\text{x}}_{{{\text{i}} 2}} {,}{\text{x}}_{{{\text{i}} 3}} {,} \ldots {\text{x}}_{iD} } \right]\quad {(}{\text{i}} = 1{,}2{,}3, \ldots {,}{\text{M}} {),} $$

10 $$ {\varvec{V}}_{id} = \left[ {v_{{i{1}}} {,}{\text{v}}_{{i{2}}} {,}v_{{i{3}}} {,} \ldots v_{iD} } \right] \quad {(}i = {1,2,3} \ldots {,}M{)}{.} $$

In each search process, there exists an optimal position for each individual and swarm, which can be expressed as:

11 $$ {\varvec{P}}_{id} = {[}{\text{p}}_{{i{1}}} {,}p_{{i{2}}} {,}p_{{i{3}}} {,} \ldots {\text{p}}_{iD} {],} $$

12 $$ {\varvec{G}}_{d} = {[}{\text{g}}_{{1}} {,}g_{{2}} {,}g_{{3}} {,} \ldots {\text{g}}_{D} {]}{.} $$

PSO can be utilized to improve the performance of the S–G filter. Firstly, the signals are filtered through the S–G filter to calculate the root mean square error (RMSE) corresponding to the individual and swarm at the current position, and then iterative optimization begins. The position and velocity are continuously updated during each iteration, while the optimal positions of the individual and swarm are adjusted by comparing the RMSE obtained before and after the update. The optimization of the window length is terminated when the maximum number of iterations is reached, and the optimal parameter of the S–G filter with fixed polynomial order is obtained.

The update equations for the velocity and position of the particle are^24^:

13 $$ \left\{ \begin{gathered} v_{id}^{{k + {1}}} = \omega v_{id}^{k} + c_{{1}} r_{{1}} {(}p_{id}^{k} - x_{id}^{k} {)} + c_{{2}} r_{{2}} {(}g_{d}^{k} - x_{id}^{k} {)} \hfill \\ x_{id}^{{k + {1}}} = x_{id}^{k} + v_{id}^{{k + {1}}} \hfill \\ \end{gathered} \right., $$

where $\omega$ is inertia weight, *c*_1_ and *c*_2_ are the learning coefficients for the particle and swarm, respectively, *c* = 0.5–2.5, ${\text{r}}_{i} {(}{\text{i}} = 1{,}2{)}$ is random number in the interval [0,1], $\omega {\text{v}}_{id}^{k}$ is the inertia of the previous behavior of the particle, ${\text{c}}_{1} {\text{r}}_{1} {(}{\text{p}}_{id}^{k} - {\text{x}}_{id}^{k} {)}$ is the cognition of the particle, ${\text{c}}_{2} {\text{r}}_{2} {(}g_{d}^{k} - {\text{x}}_{id}^{k} {)}$ is the social component.

**Figure 2:**
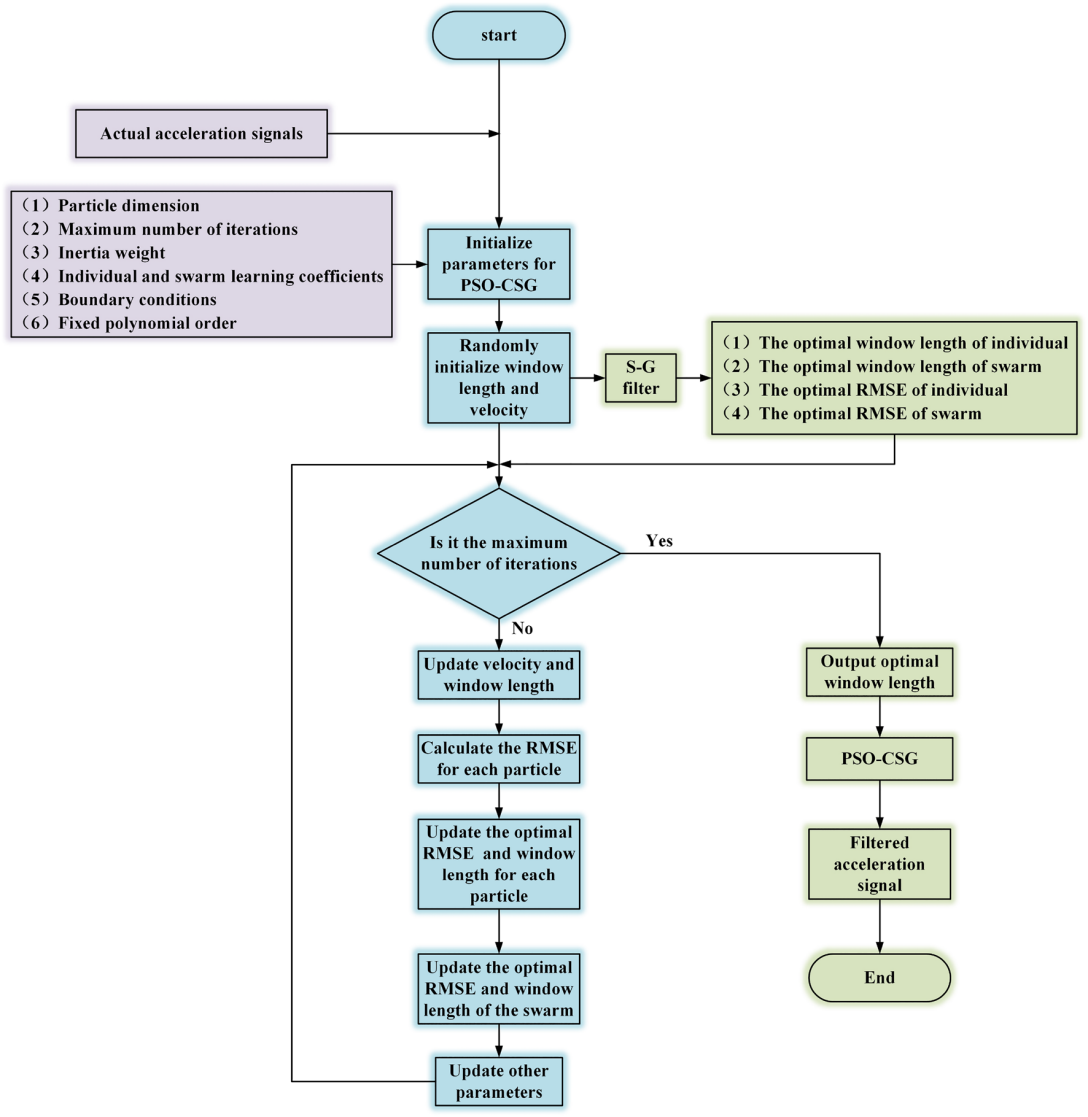
Schematic diagram of PSO-CSG.

In order to further improve the denoising effect, the acceleration signals are filtered several times by PSO-CSG. The schematic diagram of PSO-CSG is shown in Fig. 2.

#### Simulation and verification

**Figure 3:**
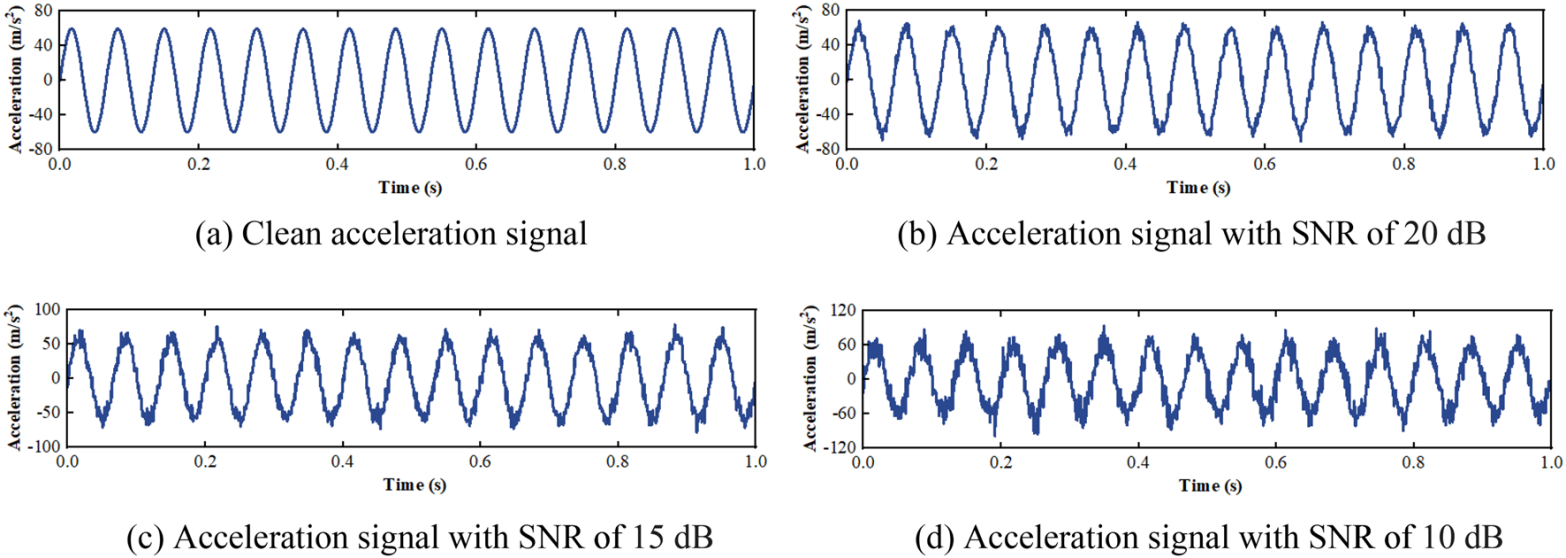
Acceleration signal with different SNR.

Assume a sinusoidal signal with frequency of 15 Hz, acceleration amplitude of 60 m/s^2^, and sampling frequency of 1000 Hz. Gaussian white noise with signal-to-noise ratio (SNR) of 10 dB, 15 dB, and 20 dB are added to the signal to simulate the interference components, respectively, as shown in Fig. 3. The clean and interference-laden acceleration signals can be expressed as:

14 $$ a_{0} (t) = 60\;\sin (30\pi t), $$

15 $$ a_{i} (t) = 60\;\sin (30\pi t) + n_{i} (t)\quad (i = 1,2,3), $$

where $n_{i} {(}t{)}$ is the Gaussian white noise with mean of 0 and standard deviation of 1.

**Figure 4:**
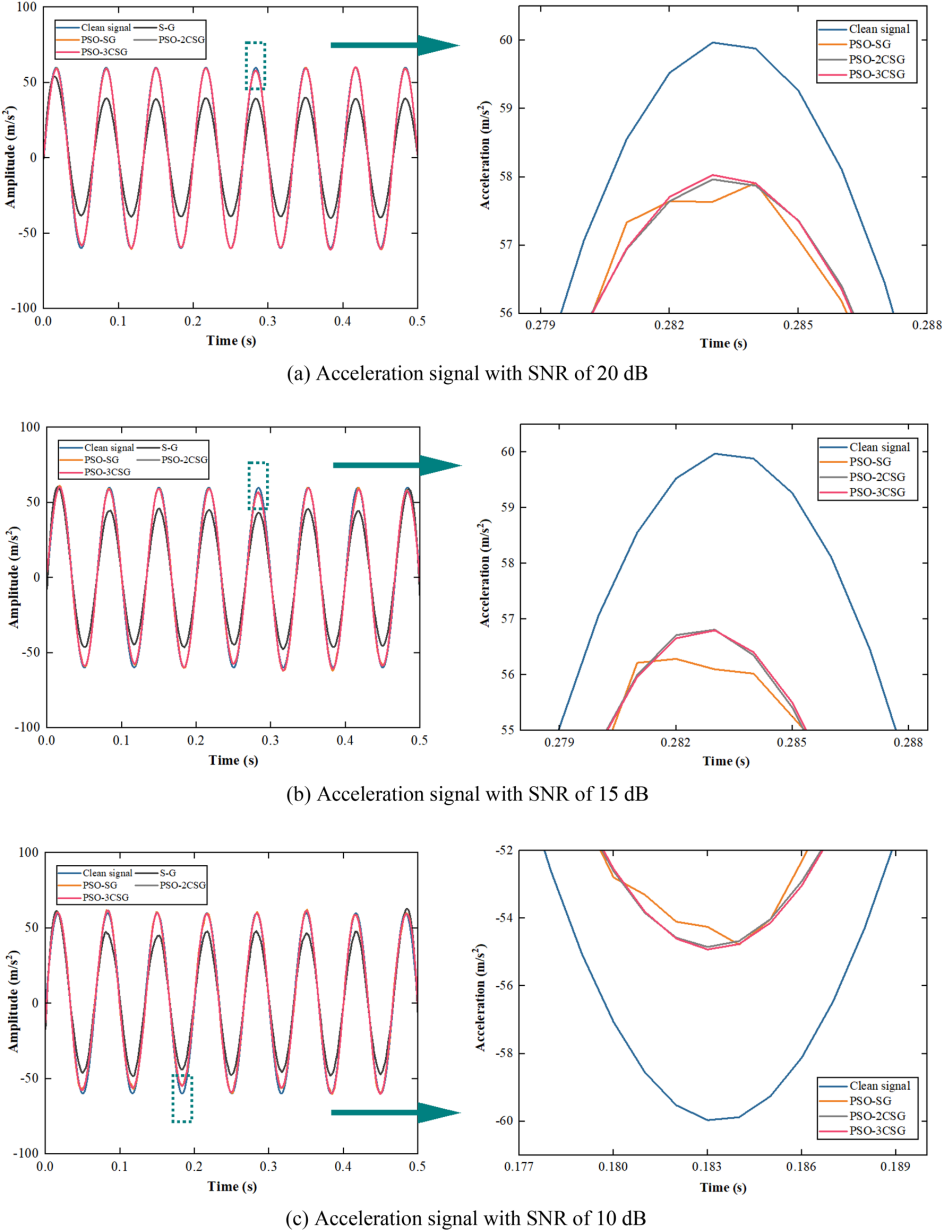
Comparison diagram of filtering effect of S–G, PSO-SG, and PSO-CSG.

The acceleration signals with different SNRs are filtered using the S–G filter, PSO-SG filter and PSO-CSG filter, and the denoising effects are compared as shown in Fig. 4.

**Figure 5:**
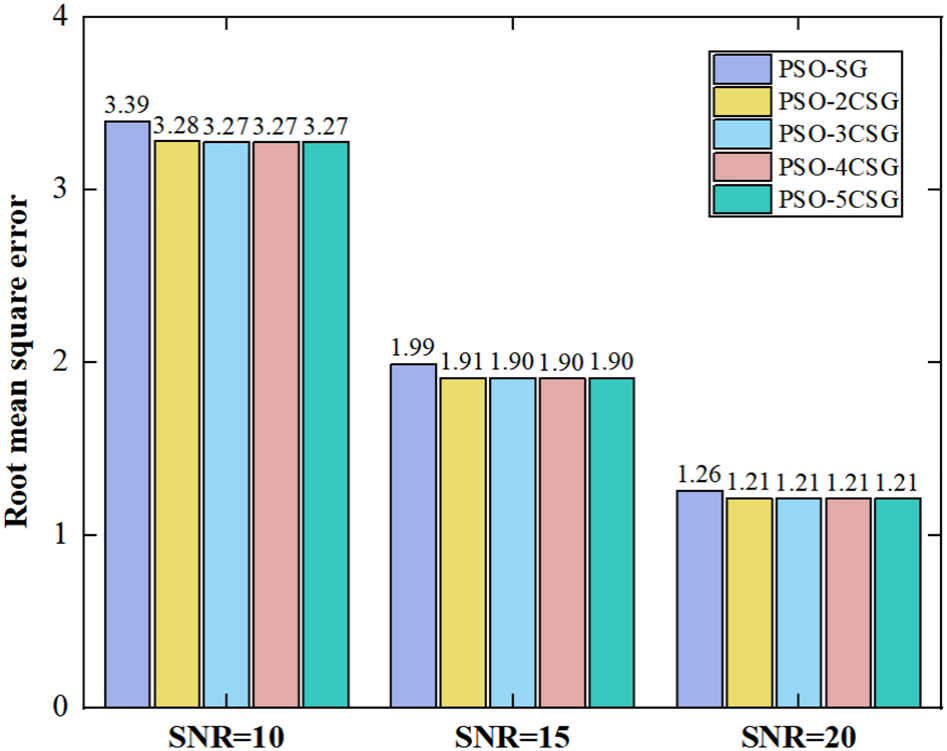
RMSE varying with SNR and the number of the cascade stage.

It can be qualitatively determined from Fig. 4 that the S–G filter is more effective at both ends of the signals compared to other regions. This is because the boundary effect of the filter results in fewer data points in the window at the signal edge. The middle part of the signals is overly smoothed through the S–G filter since the selection of window length is heavily influenced by human intervention, and the points in the signals with rapid changes and fluctuations are fitted. The filtering effect of the PSO-SG filter is better than that of the S–G filter. The main reason is that the window length is iteratively optimized with the goal of minimizing RMSE, which attenuates the subjectivity of parameter selection. The denoising effect of filters with different cascade stages is shown in Fig. 5.

It can be concluded that the PSO-SG filter performs better after cascading, and as the number of the stages of the cascade increases, the RMSE decreases. Because the smoothing effect of the filter is superimposed, and the high-frequency components of the signals can be further suppressed by multiple filtering. The RMSE stabilizes when the number of the stages of the cascade reaches 3. The performance was improved by 3.83% relative to PSO-SG. Therefore, the 3-stage cascaded filter is selected for denoising to improve effect.

### Trend item removal

#### SPA

SPA is a nonlinear method for removing the trend item based on regularization principles, commonly used in fault diagnosis and human electrocardiogram (ECG) signals processing^25^. The method has low computational complexity and can quickly separate the trend item and periodic in the signals^26^. The steps for removing the trend item from the acceleration signals using SPA are as follows:

The denoised signal is expressed as:

16 $$ {\text{a}}_{{m}} {(}t{)} = a_{{r}} {(}t{)} + {\text{a}}_{{t}} {(}t{) = }a_{{r}} {(}t{)} + W\hat{\theta }_{{\gamma }} , $$

where ***W*** is observation matrix, $\hat{\theta }_{{\gamma }}$ is optimal solution for regression parameter.

17 $$ \hat{\theta }_{{\gamma }} = {(}{\varvec{W}}^{T} {\varvec{W}} + \gamma^{2} {\varvec{W}}^{T} {\varvec{G}}_{2}^{T} {\varvec{G}}_{2} {\varvec{W}}{)}^{ - 1} {\varvec{W}}^{T} {\text{a}}_{m} , $$

where ${\gamma }$ is regularization parameter, ${\varvec{G}}_{2}$ is the discretized 2-order differential operator matrix.

The observation matrix is represented by an identity matrix to simplify the analysis. The ideal acceleration signal without the trend item is:

18 $$ \hat{a}_{{r}} = {\text{a}}_{m} - {\varvec{W}}\hat{\theta }_{{\gamma }} = \left[ {{\varvec{I}} - {(}{\varvec{I}} + \gamma^{2} {\varvec{G}}_{2}^{T} {\varvec{G}}_{2} {)}^{ - 1} } \right]{\text{a}}_{m} = {\varvec{L}}{\text{a}}_{m} , $$

where ***I*** is the identity matrix, ***L*** is a time-varying finite-impulse response high-pass filter. A detailed derivation of the result can be found in Ref.^27^.

**Figure 6:**
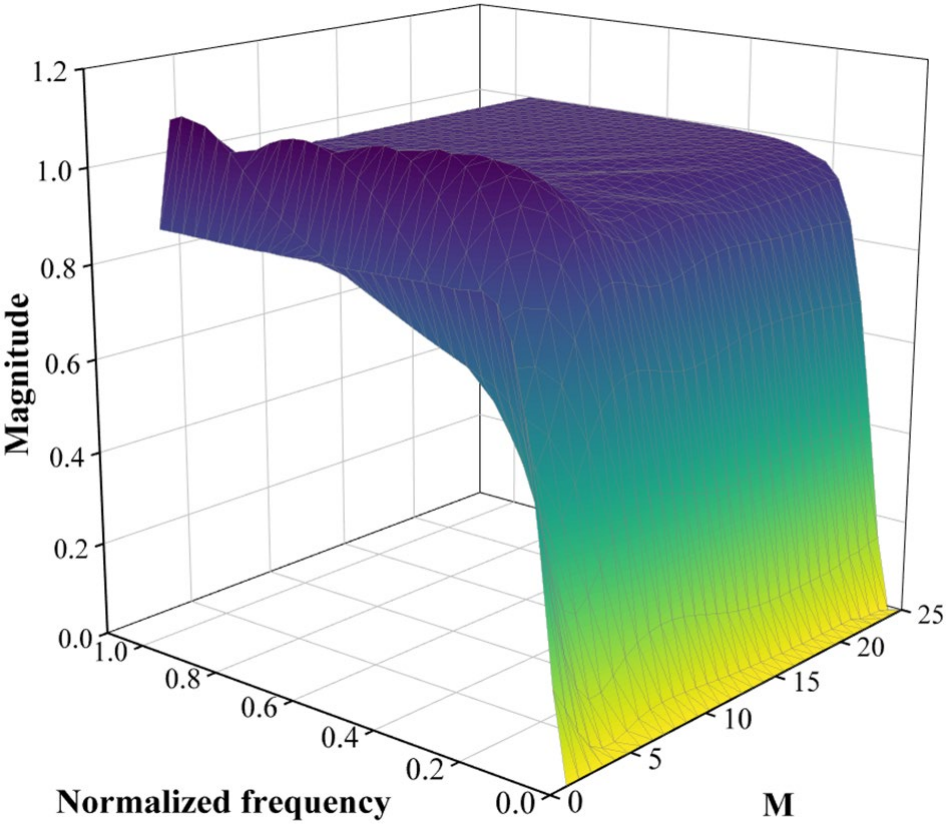
Time-varying frequency response of ***L.***

The sample size M of smoothed data is 50 and the regularization parameter is 10. The frequency characteristics of this matrix can be obtained by applying the Fourier transform to any row of the ***L***, as shown in Fig. 6.

Only the first half of the frequency response is presented due to the symmetry. It can be obtained from Fig. 6 that there is the slight fluctuation in the frequency response curve at the beginning and end of the signal. The main reason is that differential operators are introduced to capture the rate of change in the signal when using SPA. The differential operators cannot be fully applied due to the lack of adjacent data points at both ends of the signal, resulting in inaccurate estimation and boundary effect. The vast majority of the frequency response curve is smooth because there are enough data points in the signal for the differential operator to compute the rate of change.

**Figure 7:**
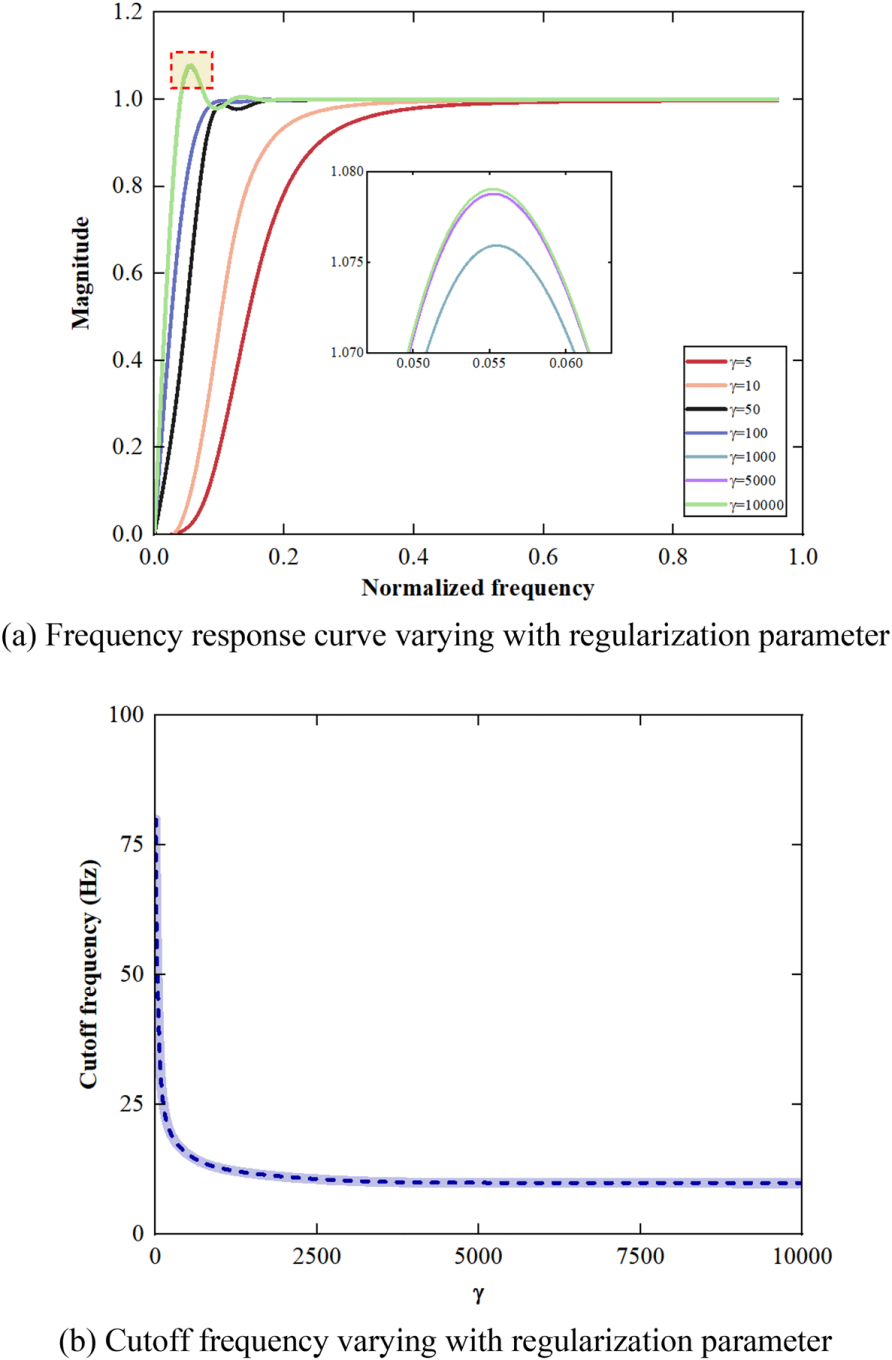
Relationship between the regularization parameter, normalized frequency and cutoff frequency.

The Fourier transform is applied to *M*/2 lines of *L* for different values of the regularization parameter ${\gamma }$. The relationship between the regularization parameters, normalized frequency and cutoff frequency is obtained as shown in Fig. 7.

As shown in Fig. 7, the normalized cutoff frequency decreases with the increase of the regularization parameter and then stabilizes. When the regularization parameter is 5000, the cutoff frequency is 9.905 Hz. The trend item of the signals can be effectively removed since the frequency range of the trend item in the acceleration signals of the vibrating screen is generally 0–10 Hz.

#### Simulation and verification

Linear, polynomial, exponential, and power trend items common in engineering are added to the clean acceleration signal, which are as follows^28^:

19 $$ {\text{tr}}_{1} {(}{\text{t}} {)} = {\text{c}}_{0} + {\text{c}}_{1} {\text{t}} , $$

20 $$ {\text{tr}}_{2} {(}{\text{t}} {)} = {\text{c}}_{0} + {\text{c}}_{1} {\text{t}} + {\text{c}}_{2} {\text{t}}^{2} + \cdots + {\text{c}}_{n} t^{n} , $$

21 $$ {\text{tr}}_{3} {(}t{)} = c_{{0}} e^{{ - c_{{1}} t}} , $$

22 $$ {\text{tr}}_{4} {(}t{)} = c_{{0}} t^{{c_{{1}} }} , $$

where the parameters ${\text{c}}_{0}$ and ${\text{c}}_{1}$ of linear trend item are 10 and − 8, respectively. The degree of polynomial trend item is 3, and the parameters ${\text{c}}_{0}$, ${\text{c}}_{1}$, ${\text{c}}_{2}$ and ${\text{c}}_{3}$ are 2, 3, 7 and − 4, respectively. The parameters ${\text{c}}_{0}$ and ${\text{c}}_{1}$ of exponential trend item are − 3 and 1.2, respectively. The parameters ${\text{c}}_{0}$ and ${\text{c}}_{1}$ of power trend item are 4 and 3, respectively.

**Figure 8:**
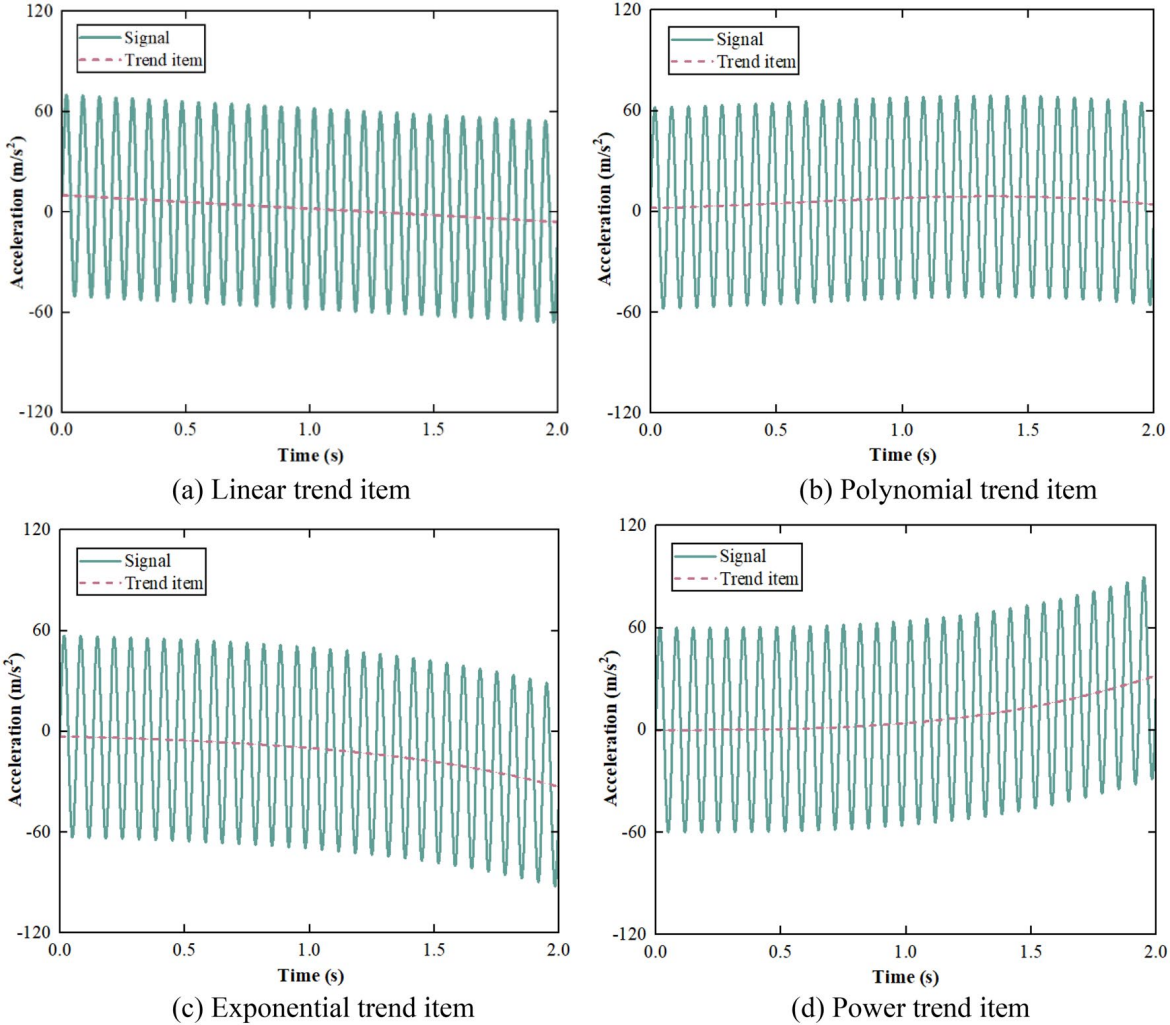
Acceleration waveform varying with trend item.

The acceleration waveform varying with the trend item is shown in Fig. 8.

**Figure 9:**
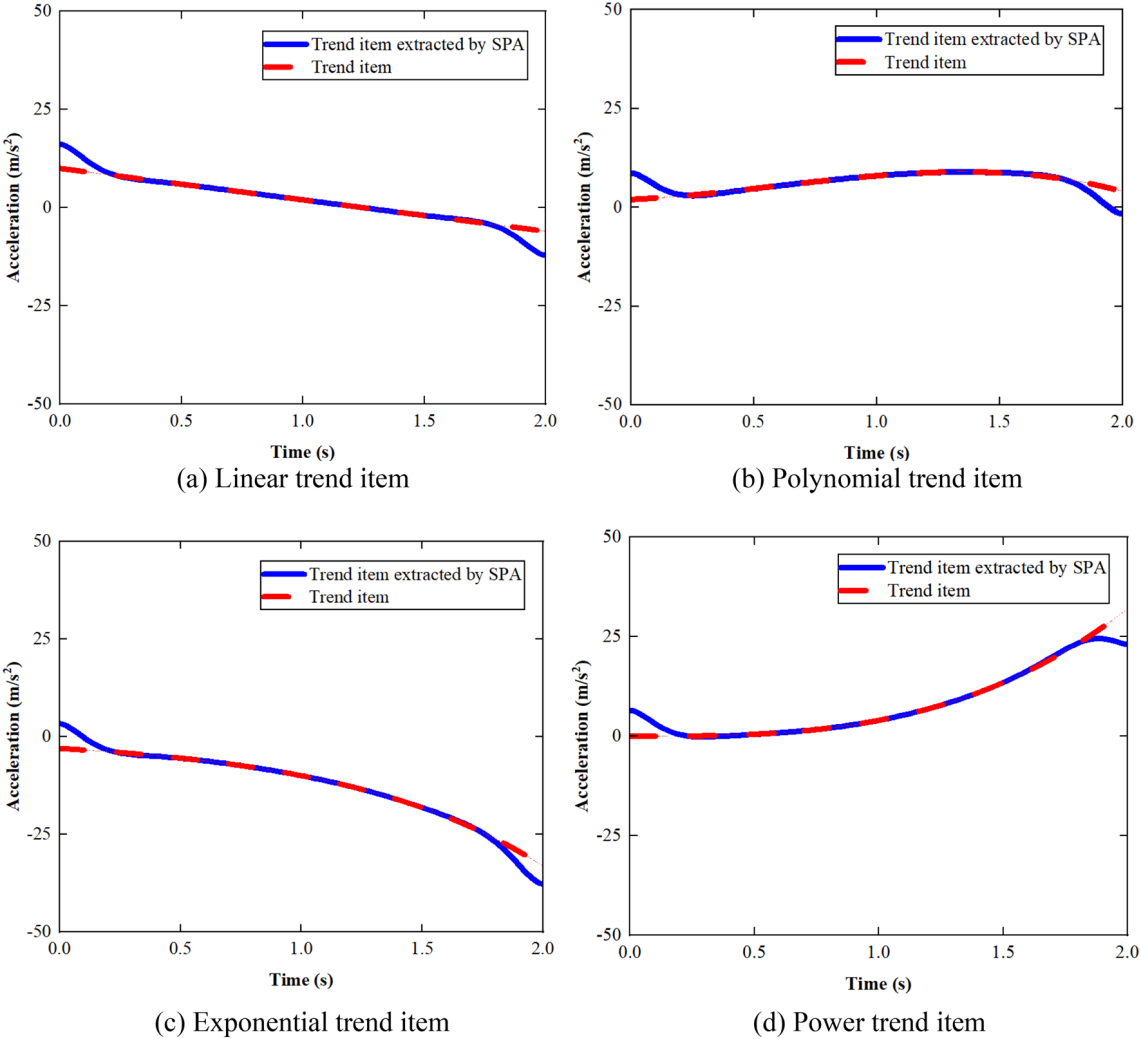
Comparison between extracted trend item and simulated trend item.

The trend item extracted using SPA is compared with the simulated trend item in Fig. 9.

It can be obtained from Fig. 9 that SPA can automatically determine and extract various types of trend items. There are errors in the extraction of the trend term at two ends due to the boundary effect.

**Figure 10:**
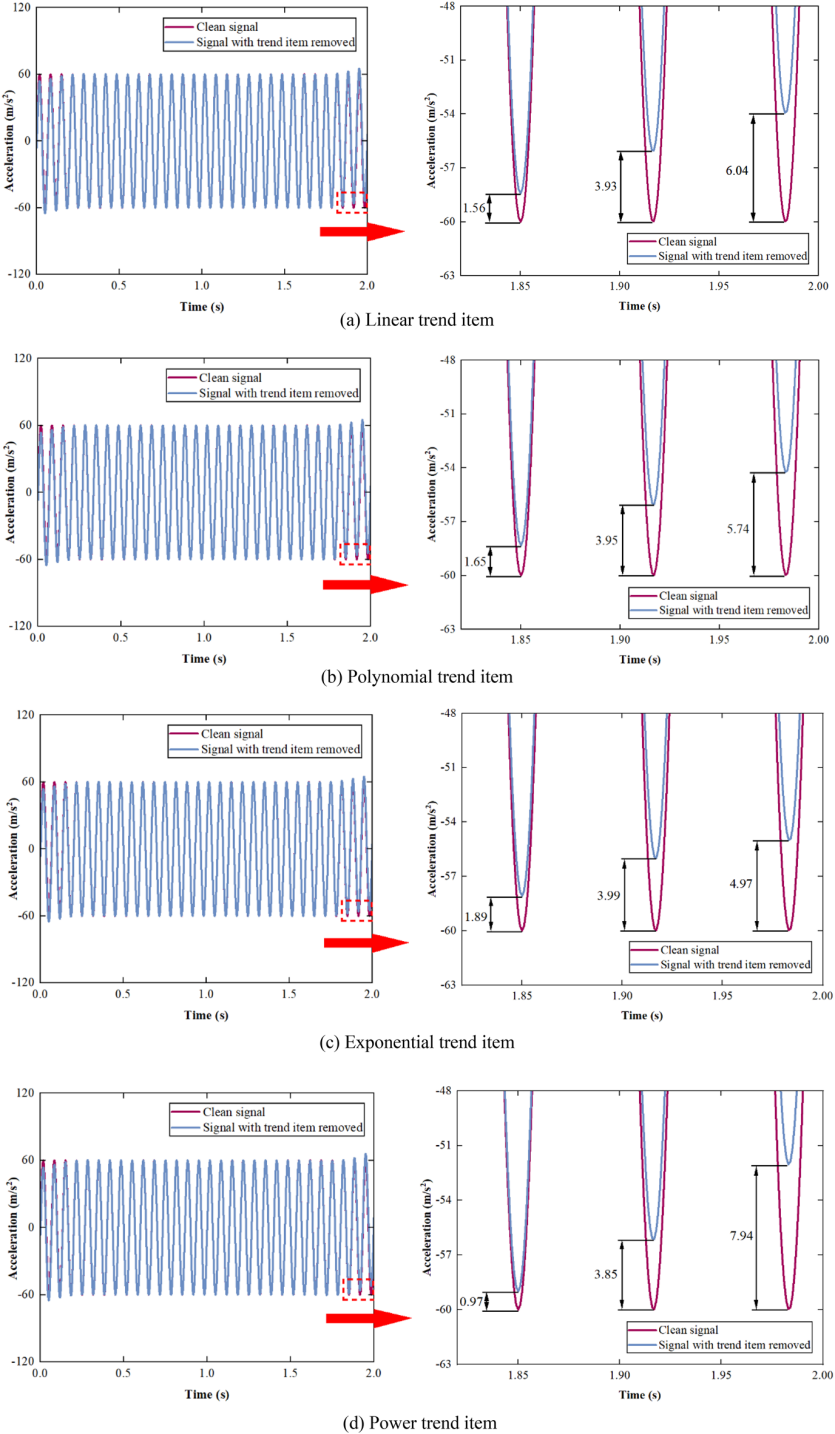
Comparison between the clean signal and the signal with trend item removed.

The comparison between the clean signal and the signal with the trend item removed is shown in Fig. 10.

**Figure 11:**
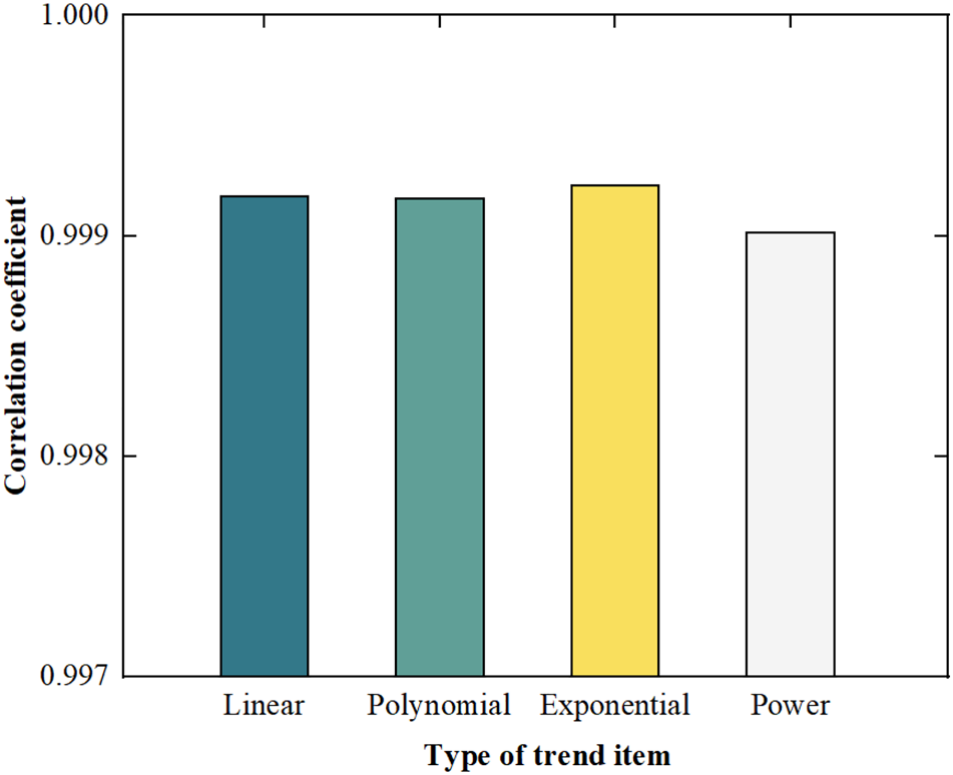
Correlation coefficient between the clean signal and the signal with trend item removed.

As shown in Fig. 10, the boundary effect still occurs at both ends of the acceleration signals after removing the trend term due to the transmissibility of the linear operation, which has the greatest impact on the extreme points. The closer the positions are to the edge of the data, the greater the impact is. However, as shown in Fig. 11, the correlation coefficients between the acceleration signals with the trend term removed and the clean acceleration signal are 0.99918, 0.99917, 0.99923, and 0.99902, respectively. The boundary effect has a relatively small impact on the overall signals and can be ignored in practical engineering. Therefore, the SPA is very effective in removing the trend item from the signals.

## Frequency-domain integration

Frequency-domain integration is a method of signal processing and analysis used to process signals in the frequency domain^29^. The preprocessed time-domain acceleration signal is discretized by discrete Fourier transform to obtain the spectrum:

23 $$ {\text{a}}_{{r}} {(}k{)} = \left\{ {{(}a_{{0}} {,}b_{{0}} {\text{j}} {),(}a_{{1}} {,}b_{{1}} {\text{j}} {),} \ldots {,(}a_{{{\text{N}} - {2}}} {,}b_{{{\text{N}} - {2}}} {\text{j}} {),(}a_{{{\text{N}} - {1}}} {,}b_{{{\text{N}} - {1}}} {\text{j}} {)}} \right\}, $$

where *N* is length of the discrete acceleration sequence,* j* is imaginary number.

The displacement signal in the frequency domain can be derived by double integrating and superposing each harmonic of acceleration, which is expressed as^30^:

24 $$ s(t) = \sum\limits_{k = 0}^{N - 1} {A_{sk} \;\cos (\omega_{sk} t + \varphi_{sk} )} , $$

where ${\text{A}}_{sk} = \frac{{\sqrt {{\text{a}}_{\text{k}}^{2} + {\text{b}}_{\text{k}}^{2} } }}{{\omega_{\text{k}}^{2} }}$ is amplitude, $\omega_{sk} = 2\pi \frac{k}{\text{N}}$ is circular frequency, $\varphi_{sk} = \arctan \frac{{b_{k} }}{{a_{k} }} - \pi$ is phase angle.

The displacement signal in the time domain is obtained using inverse Fourier transform, which can be expressed as follows:

25 $$ {\text{s}} {(}t{)} = - F^{{ - {1}}} \left[ {\frac{{1}}{{\omega^{2} }}F{[}a{(}t{)]}} \right]{.} $$

## Test verification

The vibration acceleration signals of the mining vibrating screen are difficult to measure limited by test conditions. The vibration test platform is built in order to verify the correctness of the theoretical model proposed in this paper. The maximum vibration frequency of the test platform is 25 Hz, and the range of double amplitude is 3–15 mm. The vibration frequency of the mining vibrating screen studied by most scholars is about 16 Hz with the double amplitude between 8 and 9 mm^31^, but many mining vibrating screens with different vibration frequencies and displacements that have been applied in actual production^32^. Therefore, the test platform can meet the test requirements.

### Vibration test platform

**Figure 12:**
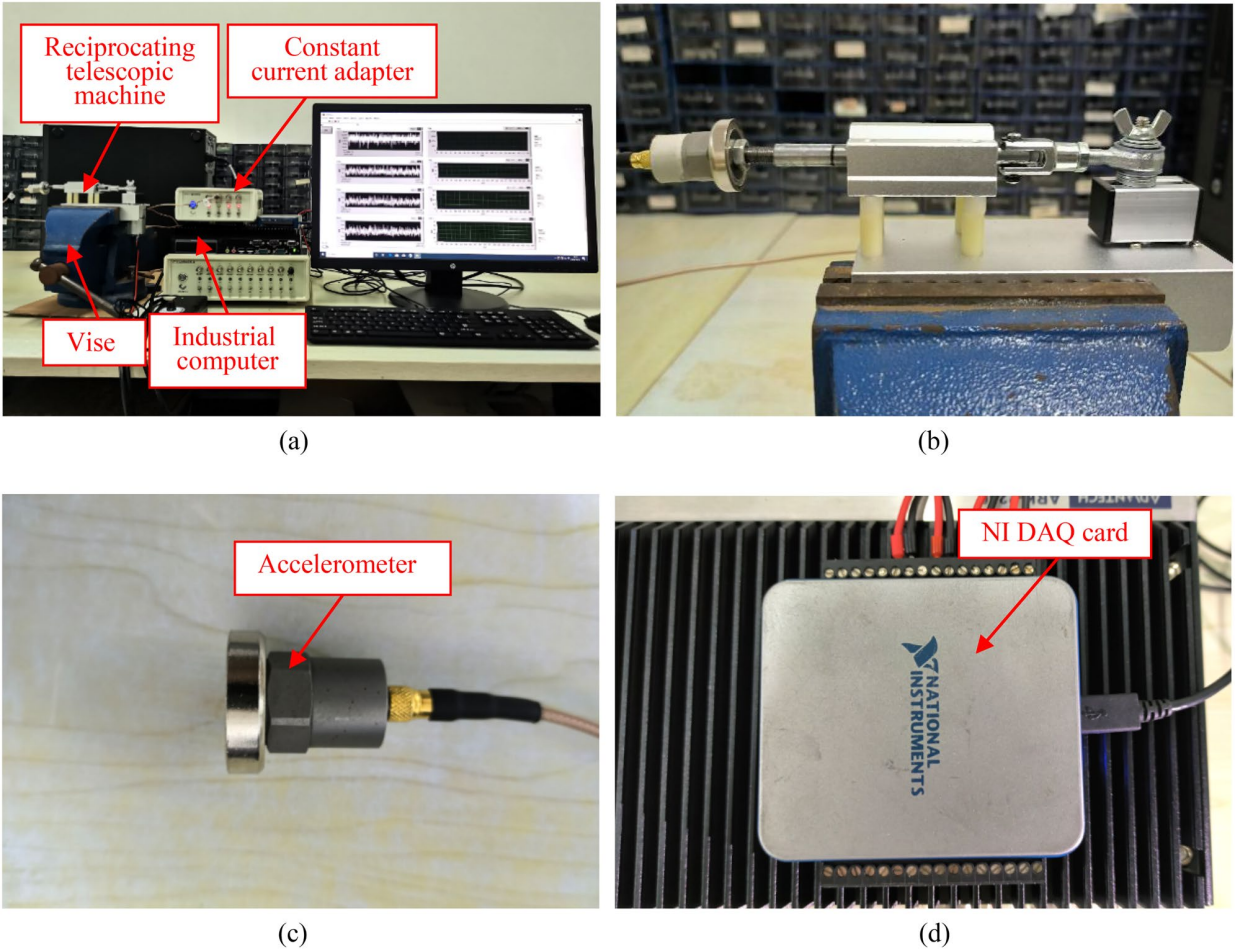
Vibration test platform.

The test platform consists of a reciprocating telescopic machine, piezoelectric accelerometer, NI DAQ card, constant current adapter, and an industrial computer as shown in Fig. 12. The reciprocating telescopic machine is fixed with a vise in the test. The model of the acceleration sensor is CT1010L, which is installed at the end of the top rod of the reciprocating telescopic machine to measure the vibration voltage signals of the test platform, and the parameters of the accelerometer are shown in Table 1. The accelerometer is connected to the constant current adapter, and the measured voltage data is transmitted to the industrial computer through the NI DAQ card. The voltage signals are converted to acceleration signals, the waveforms are plotted, and the data is stored by LabVIEW.

**Table 1:** Parameters of the accelerometer.

Parameters	Value
Model	CT1010L
Acceleration range/(g)	± 50
Frequency response/(Hz)	1–10 k
Voltage sensitivity/(mV/g)	100
Resolution ratio/(g)	0.0005
Operating temperature range/(℃)	− 20 to 120
Operating voltage/(V)	18–30

The sampling frequency of the accelerometer is set to 1000 Hz according to Nyquist’s sampling theorem. During the measurement process, the test data collection is carried out after the vibration mechanism is turned on and running for a period of time.

### Sampling frequency analysis

**Figure 13:**
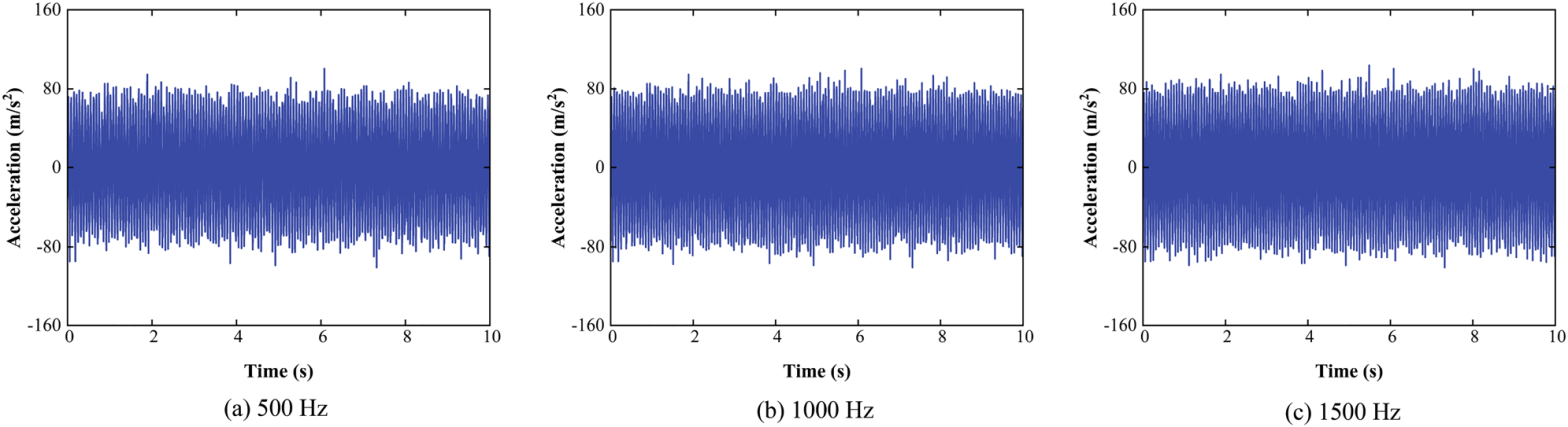
Signals with different sampling frequencies.

In order to analyze the effect of different sampling frequencies on integration error. The sampling frequencies are set to 500 Hz, 1000 Hz and 1500 Hz, respectively. The acceleration signals with different sampling frequencies are shown when the double amplitude of the reciprocating telescopic machine is 11 mm in Fig. 13.

**Figure 14:**
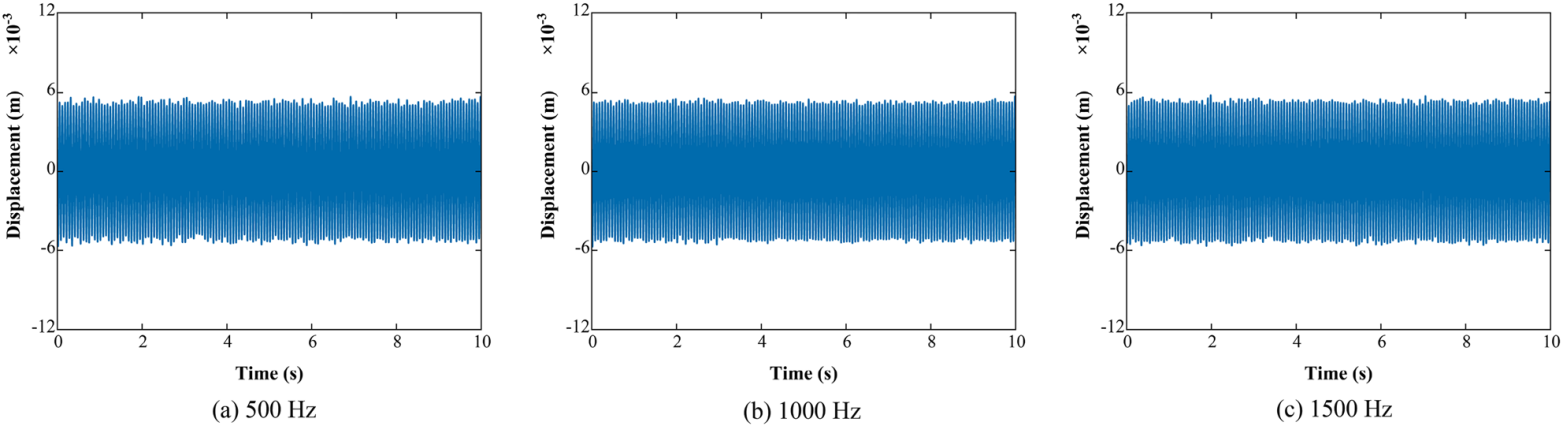
Displacement signals.

The displacement signals obtained through the proposed method are shown in Fig. 14.

**Figure 15:**
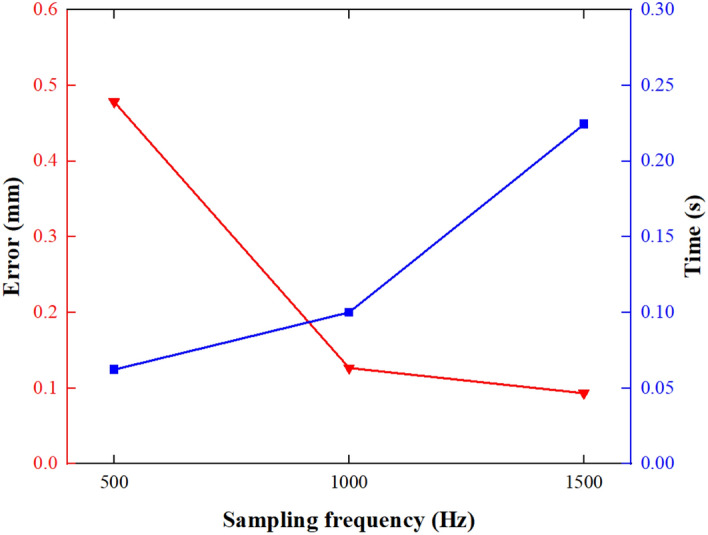
Integration error and time with different sampling frequencies.

The effect of sampling frequencies in terms of integral accuracy and efficiency is shown in Fig. 15.

It can be seen from Fig. 15 that the integral accuracy is significantly improved compared to 500 Hz when the sampling frequencies are 1000 Hz and 1500 Hz. The integration time of 1000 Hz is shorter than that of 1500 Hz. Therefore, the integral accuracy and efficiency can be ensured when the sampling frequency is 1000 Hz. The selected accelerometer is effective.

### Test results and discussion

**Figure 16:**
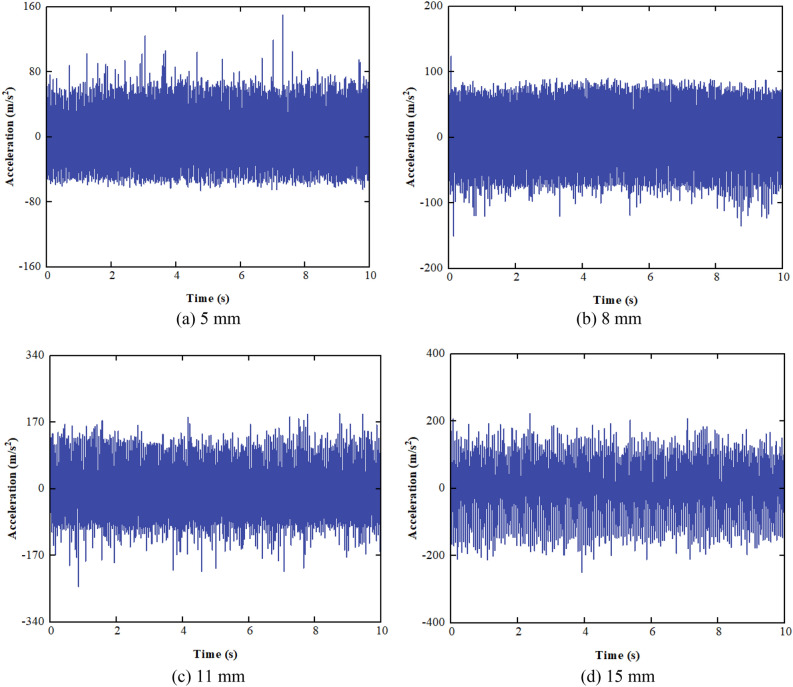
Acceleration waveform varying with double amplitude.

The test is performed at values of 5 mm, 8 mm, 11 mm, and 15 mm for double amplitude. The Gaussian white noises with powers of 202 W, 152 W, 978 W, and 651 W are added to acceleration signals, resulting in an SNR of 10 dB for all acceleration signals. The acceleration waveforms under different double amplitudes are shown in Fig. 16.

**Figure 17:**
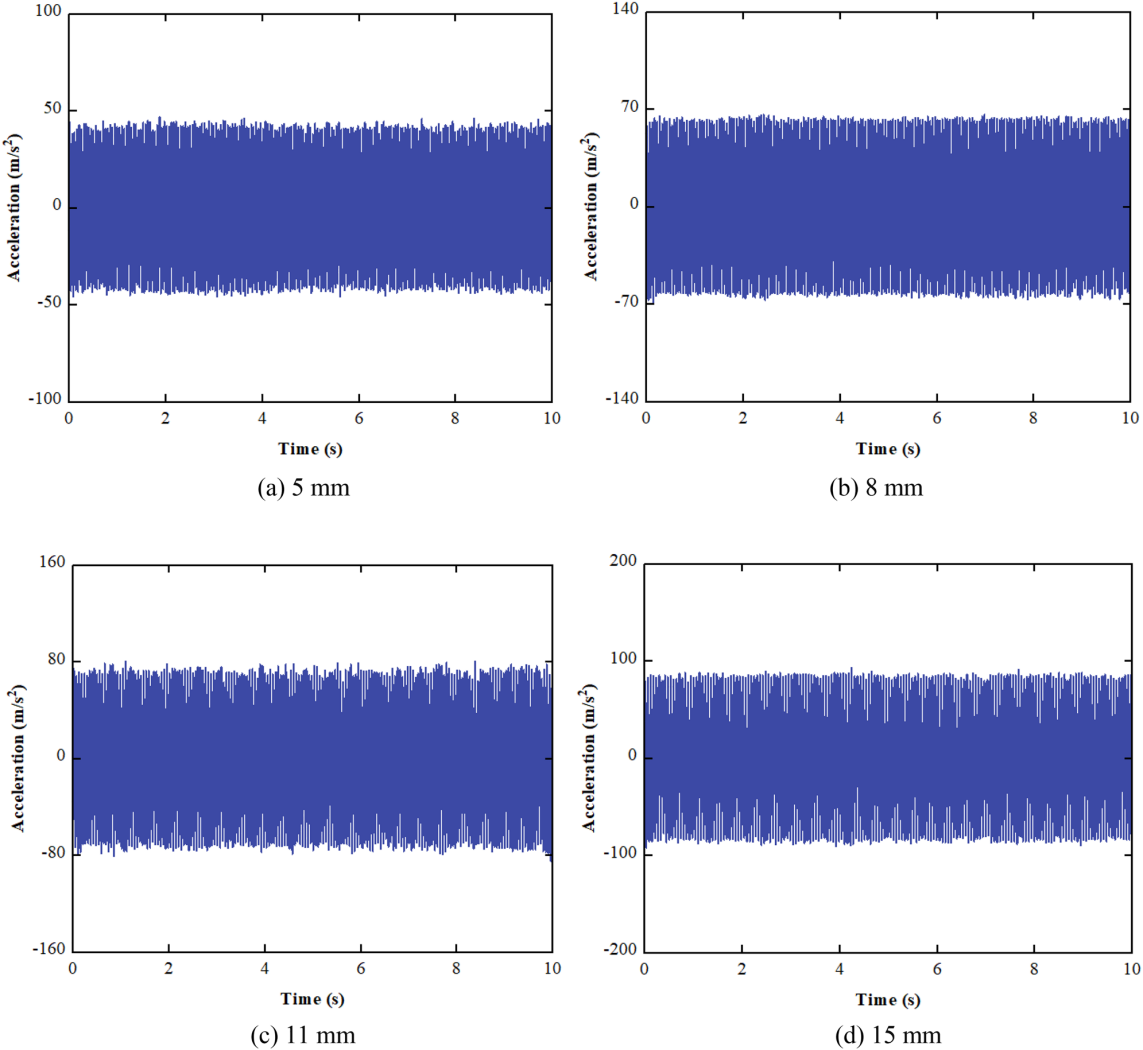
Waveform of preprocessed acceleration.

It can be seen from Fig. 16 that the SNR of the acceleration signals is low, and the effective signals are completely swamped by noise. The acceleration signals are preprocessed, including denoising and trend item removal, as shown in Fig. 17.

**Figure 18:**
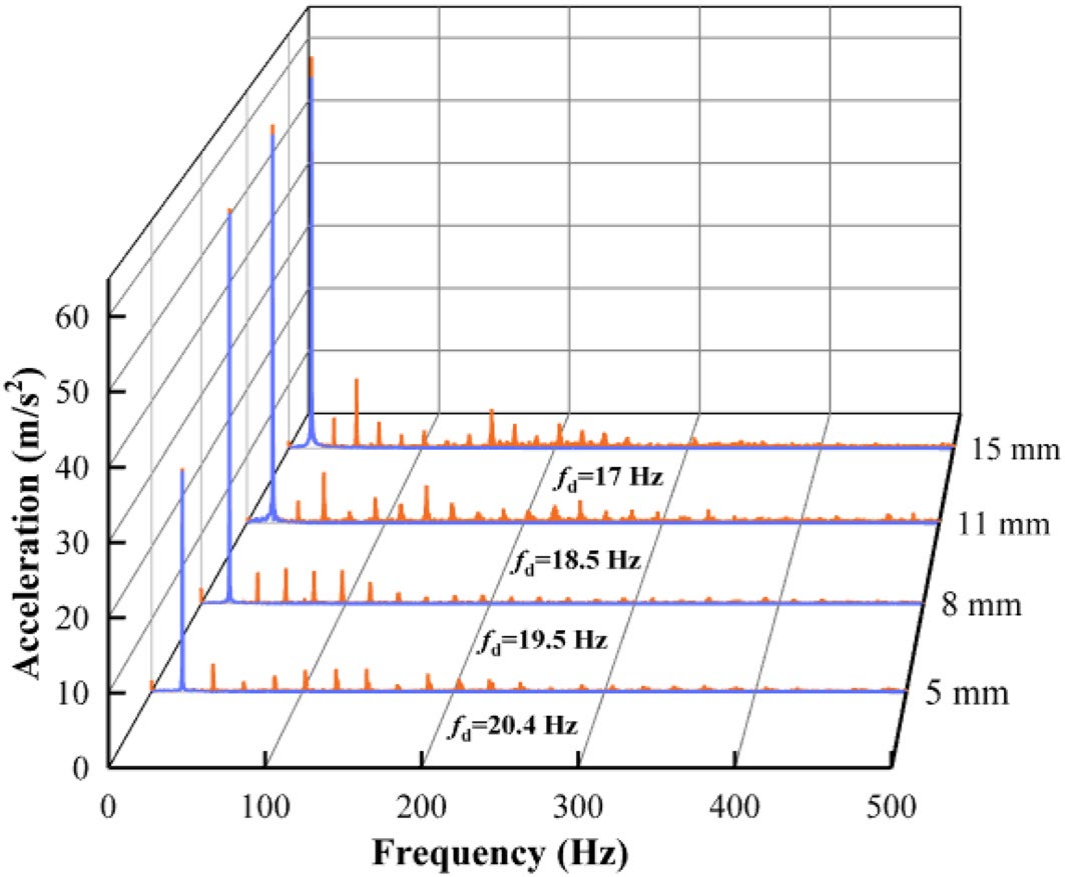
Comparison of acceleration spectra before and after preprocessing.

The comparison of acceleration spectra before and after preprocessing is shown in Fig. 18.

**Figure 19:**
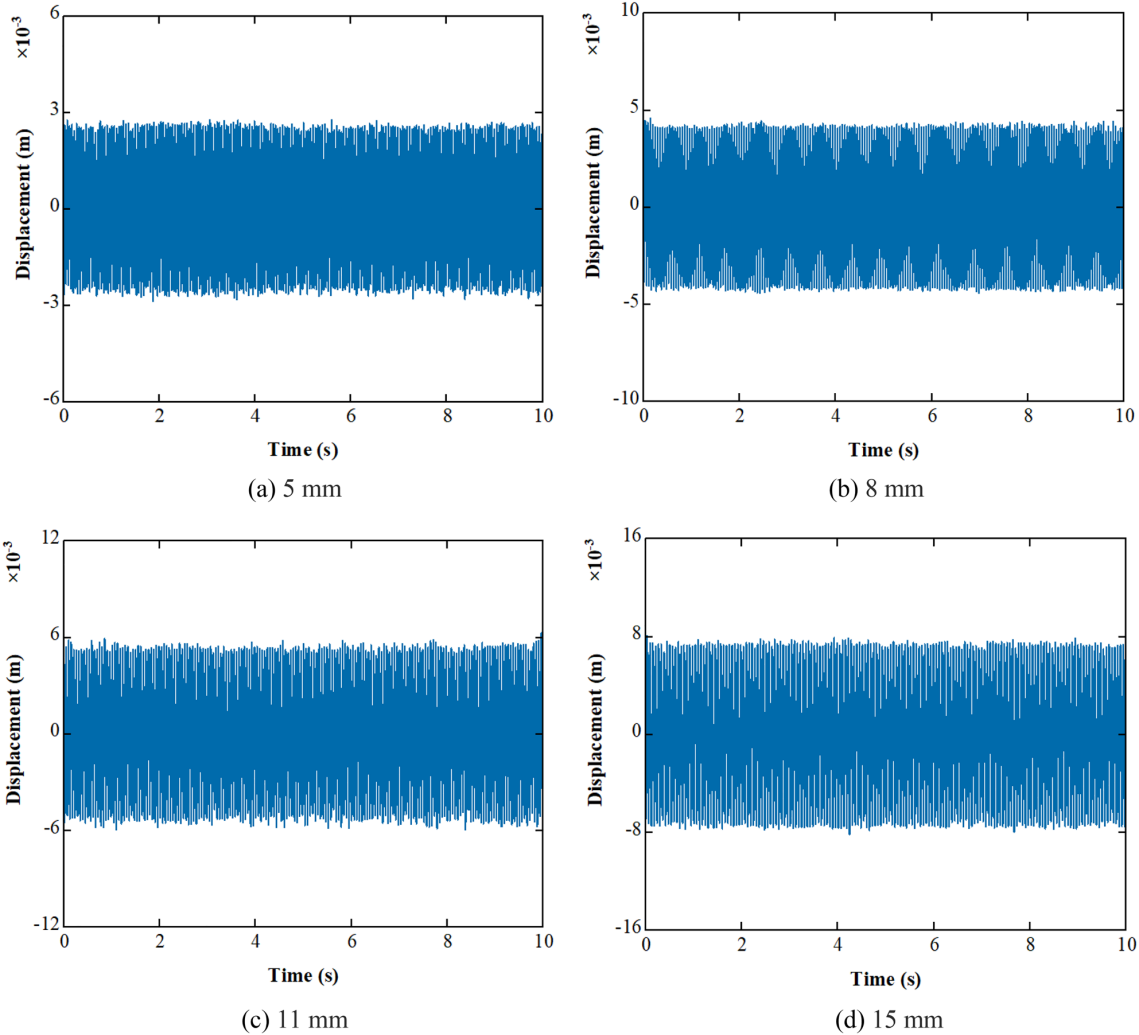
Waveform of displacement.

The *f*_d_ in Fig. 18 is the major frequency of the signals. It can be seen that the major frequencies of the signals are retained after preprocessing, and the high-frequency noise and low-frequency trend items are effectively removed. The filtering method proposed in this paper is effective in denoising and removing trend items. The displacements obtained by integrating the preprocessed signals in the frequency domain are shown in Fig. 19.

**Figure 20:**
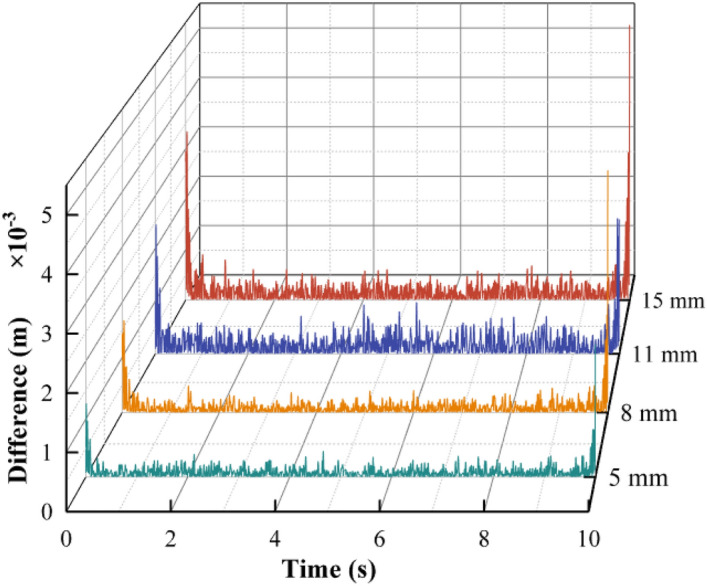
Difference between the theoretical and test displacement.

The difference between the theoretical and test displacement is shown in Fig. 20.

It can be concluded that the amplitude of the theoretical displacement differs slightly from the test displacement due to the accumulation of errors during preprocessing. However, the difference of displacement is kept within 0.3 mm, and the accuracy can meet the actual engineering requirements. The integration result of the proposed theoretical model is accurate and can be used to obtain the vibration amplitude of the mining vibrating screen.

## Conclusion

In order to obtain displacement signals of the vibrating screen accurately, the method for converting vibration acceleration to displacement based on the improved S–G filter is proposed in this paper. The main conclusions are as follows:

1. The PSO algorithm is used to optimize the window length of the S–G filter with fixed polynomial order. It is found that the window length size can be selected objectively, and the subjectivity of the traditional S–G filter in parameter selection is reduced by the improved S–G filter with significant denoising effect. The PSO algorithm has a good optimization effect, and the improved S–G filter is more suitable to denoise the vibration signals of the mining vibrating screen.
2. The effect of the number of stages of cascade on the denoising effect is studied. As the number increases, the RMSE decreases and the denoising effect of the filter improves under the same SNR. The RMSE stabilizes and the performance is improved by 3.83% relative to PSO-SG when the number reaches 3. The 3-stage cascaded filter is selected to denoise for efficiency, which has a good denoising effect under different SNR.
3. The cutoff frequency of the SPA filter decreases with the increase of the regularization parameter. When the regularization parameter reaches 5000, the cutoff frequency tends to stabilize. Different types of trend items can be effectively extracted and removed by the SPA filter. The correlation coefficients between the clean signal and the signals with the trend item removed are all above 0.9.
4. The theoretical model is verified by building the test platform. It is found that the theoretical model is effective in removing high-frequency noise and low-frequency trend items under different amplitudes. The difference between the theoretical and measured displacement can be kept within 0.3 mm. The accuracy of the method can meet engineering requirements and has high practical application value.

## Data Availability

The data used to support the findings of this study are available from the corresponding author upon request.
